# Purine Nucleoside Phosphorylase Inhibition Rebalances Purine Metabolism and Attenuates Organ Damage in Sickle Cell Mice

**DOI:** 10.1111/jcmm.70996

**Published:** 2025-12-30

**Authors:** Adekunle Emmanuel Alagbe, Lynda Little‐Ihrig, Stephanie M. Mutchler, Edwin K. Jackson, Enrico M. Novelli, Stevan P. Tofovic

**Affiliations:** ^1^ Heart, Lung, Blood and Vascular Medicine Institute University of Pittsburgh School of Medicine Pittsburgh Pennsylvania USA; ^2^ Department of Medicine University of Pittsburgh School of Medicine Pittsburgh Pennsylvania USA; ^3^ Division of Renal Electrolytes University of Pittsburgh School of Medicine Pittsburgh Pennsylvania USA; ^4^ Department of Pharmacology and Chemical Biology University of Pittsburgh School of Medicine Pittsburgh Pennsylvania USA; ^5^ Division of Classical Hematology University of Pittsburgh School of Medicine Pittsburgh Pennsylvania USA

## Abstract

Red blood cells (RBCs) contain the highest purine nucleoside phosphorylase (PNP) level per cell volume, yet the role of PNP in the pathogenesis of sickle cell disease (SCD) is incompletely understood, highlighting an important gap in our knowledge of the disease. Previously, we reported increased PNP release by RBCs and accelerated purine nucleoside metabolism with increased production of pro‐oxidant, pro‐inflammatory and vasculotoxic byproducts in children with SCD and animal models of hemolytic injury. Thus, we hypothesized that PNP inhibition would reduce hemolysis and attenuate end‐organ damage in SCD. In adult patients with SCD (*n* = 63), plasma PNP levels were markedly elevated compared to controls (*n* = 27; *p* < 0.001) and correlated positively with LDH (*r* = 0.6032, *p* < 0.0001) and negatively with haemoglobin (*r* = −0.4523, *p* = 0.0002). SCD mice also showed accelerated purine metabolism compared to controls. Treatment with the PNP inhibitor 8‐aminoguanosine (8‐AG) increased inosine and guanosine and reduced downstream vasculotoxic byproducts hypoxanthine (*p* = 0.036), xanthine (*p* = 0.004) and guanine (*p* = 0.047), indicating efficient PNP inhibition. 8‐AG treatment rebalanced the purine metabolome in SCD mice to favour protective over harmful purine metabolites without negatively affecting haematological parameters. This was associated with reduced hemolysis and decreased splenomegaly, hepatomegaly, and hepatic and renal injury. This study suggests that PNP is an important erythrocytic damage‐associated molecular pattern molecule involved in the complex pathophysiology of SCD and proposes PNP inhibitors as a new therapeutic option for SCD and other hemolytic diseases.

## 1 Introduction

Sickle cell disease (SCD) is an inherited, severely disabling disease affecting millions of people globally [1, 2] and is caused by a missense mutation in the *HBB* gene encoding the β‐globin subunit of haemoglobin [3, 4, 5]. The mutated haemoglobin in SCD (HbS) is less soluble and is prone to polymerisation upon deoxygenation leading to the deformation of red blood cells (RBCs) into rigid crescents or sickle cells that are prone to intra‐ and extravascular hemolysis. RBC alterations and hemolysis lead to a proinflammatory state that causes hyper‐adhesion of blood cells. Combined, these pathogenic mechanisms result in vaso‐occlusion, ischemic/reperfusion injury, endothelial dysfunction, and oxidative damage [6, 7, 8]. Common multi‐organ complications in SCD include recurrent pain episodes, anaemia, splenomegaly, liver and kidney dysfunction, and pulmonary hypertension [6, 7]. Acute and chronic organ damage has a profound effect on the quality of life and longevity of individuals with SCD.

Intravascular hemolysis releases erythrocyte damage‐associated molecular patterns (eDAMPs) that enhance oxidative vascular injury, inflammation and blood cell aggregation [7, 9]. In this regard, RBCs contain high micromolar amounts of ATP (a major source of adenosine production) [10, 11], as well as large amounts of adenosine deaminase (ADA) and purine nucleoside phosphorylase (PNP), two enzymes involved in purine nucleoside catabolism [12, 13, 14, 15]. We hypothesize that ATP, ADA and PNP may comprise a critically important set of eDAMPs released by damaged RBCs in patients with SCD (see Graphical Overview). In support of the eDAMP hypothesis, studies show that recurrent hemolysis increases intravascular ATP [16, 17], which is rapidly degraded to adenosine [10, 11]. Adenosine is efficiently taken up by RBCs or converted to inosine by ADA, thus releasing ammonium (NH_4_), which can trigger a cascade of inflammatory reactions [18]. Previously, we reported increased systemic PNP and ADA activities in children with SCD and animals exposed to hemolytic insult [13, 14, 15]. PNP converts inosine to pro‐oxidative hypoxanthine leading to endothelial dysfunction [19], and xanthine oxidase (XO) converts hypoxanthine to xanthine with the generation of peroxide and superoxide [20]. PNP also catalyses the conversion of guanosine to guanine, and guanine deaminase (guanase, GDA) catalyses the hydrolytic deamination of guanine [21], resulting in the production of vasculotoxic xanthine [22] and pro‐inflammatory NH_4_. Notably, inosine and guanosine have well‐documented antioxidant [23, 24, 25, 26], anti‐ischemic [27, 28, 29, 30], anti‐inflammatory [24, 31, 32], antiplatelet/antithrombotic [33, 34] and antinociceptive effects [35, 36, 37, 38] that could be protective and beneficial in SCD. The increased PNP activity and accelerated purine nucleoside metabolism may abolish the protective effects of inosine and guanosine, whereas PNP inhibition may potentiate their beneficial effects in SCD [39]. Also relevant to SCD, in a murine model of chronic pain [40], the PNP inhibitor forodesine potentiates the antinociceptive effects of inosine [36]. Together, these findings corroborate our hypothesis that in SCD, the systemic actions of PNP and ADA are increased, which eventually results in accelerated purine nucleoside metabolism with subsequent depletion of tissue‐protective purines and simultaneous accumulation of tissue‐damaging purines.

The above considerations motivate our hypothesis that modulating purine metabolism by inhibiting PNP activity would alter purine metabolism balance towards protective and away from harmful purines and thereby prevent or ameliorate organ damage in SCD [39]. For the first time, we demonstrate here that inhibition of PNP by 8‐aminoguanosine (8‐AG) restores the purine metabolism balance in SCD, resulting in reduced hemolysis and end‐organ damage in SCD mice. Thus, PNP inhibition could potentially be a new therapeutic strategy in SCD and other hemolytic diseases.

## 2 Methods

For a detailed description of the materials and methods, see Appendix S1.

### 2.1 Human Plasma Samples

We obtained banked citrated plasma samples collected from patients with SCD (*n* = 63) during steady state, as well as race, age and sex‐matched HbAA controls (*n* = 27) enrolled in a prior clinical trial (NCT02946905). All participants were of African descent. Among the patients with SCD, 38 had severe disease (HbSS and HbS/β^0^ thalassaemia) and 25 ‘mild’ disease (HbSC and HbS/β^+^ thalassaemia). See Appendix S1 for details of the clinical parameters of the participants. The patients' clinical laboratory data were obtained at the time of their clinic visit. The frozen plasma samples were then batch tested for PNP by ELISA according to the manufacturer's protocol.

### 2.2 Mice

Humanised transgenic Townes AA mice (Hba^tm1(HBA)Tow^ Hbb^tm3(HBG1,HBB)Tow^) and SS mice (Hba^tm1(HBA)Tow^ Hbb^tm2(HBG1,HBB*)Tow^) have been previously described [40, 41, 42]. Six to seven‐week‐old male AA and SS mice were obtained from Jackson Laboratory, and the University of Pittsburgh Animal Care and Use Committee (IACUC) approved the animal use protocol. At 10 weeks of age, the AA and SS mice were randomised to receive 8‐AG (80 mg/kg/day) in drinking water (AA+8‐AG group, *n* = 9 and SS + 8‐AG group, *n* = 11) or had *ad libitum* access to tap water (AA group, *n* = 9 and SS group, *n* = 11) for 20 weeks. An additional subset of 10‐week‐old male SS mice was treated with 8‐AG (60 mg/kg/day) in drinking water (SS + 8‐AG group *n* = 11) or consumed tap water (SS group, *n* = 11) for 8 weeks.

### 2.3 Metabolic Cage Studies

#### 2.3.1 Urine Collection

At predetermined time intervals, animals were placed in a metabolic cage and allowed to acclimatise for at least 16 h, and urine samples were collected over 24 h. Urine samples were centrifuged at 10,000 ***g*** for 10 min at 4°C, sediments discarded and supernatants stored at −80°C until analysis.

#### 2.3.2 Blood Collection

Blood samples were collected retro‐orbitally at given time points from each mouse under isoflurane anaesthesia using a heparinized microcapillary tube or terminally by syringe via cardiac puncture. Blood samples were transferred to an EDTA tube and immediately analysed using the HEMAVET HV950 auto‐analyser (Drew Scientific, Plantation, FL). The remaining blood was centrifuged at 2500 ***g*** for 10 min at 4°C and, separated RBC pellets and plasma were stored at −80°C until analysis.

### 2.4 Urinary Metabolome

To determine 8‐AG metabolism and to investigate the effects of PNP inhibition on purine metabolome, urine samples were analysed for urinary adenosine, inosine, guanosine, guanine, hypoxanthine, xanthine, 8‐AG, and 8‐aminoguanine using ultra‐high‐performance liquid chromatography–tandem mass spectrometry (UPLC‐MS/MS), as previously described [43].

### 2.5 Biochemical Analysis

Plasma haemoglobin and AST, urinary haemoglobin, creatinine and albumin, and liver iron content were determined using colorimetric assays or ELISA kits according to manufacturers' protocols. The kits and reagents used are listed in Table S1.

### 2.6 Closed Chest Right Ventricle Catheterization and Transthoracic Echocardiography of the Left Ventricle

Closed chest catheterization for in vivo right ventricle (RV) pressure measurements was performed at the University of Pittsburgh Vascular Medicine Institute (VMI) Small Animal Hemodynamic Core Lab, based on a previously documented protocol [44, 45] and as described in Appendix S1. Non‐invasive transthoracic echocardiography of the left ventricle (LV) was performed at the University of Pittsburgh VMI Echocardiography Core using the Vevo 3100 ultrasound machine (FUJIFILM Visual Sonics, Toronto, Canada) as previously described [41, 46]. Focus was on the left heart, as the non‐invasive echo of the right ventricle has a limited visual window and inconsistent reproducibility (details are in Appendix S1).

### 2.7 Tissue Processing and Histopathology

At the end of each treatment period, the mice were euthanized, and the organs were immediately removed and processed as follows The organs were weighed and bisected or divided into two groups—one was snap‐frozen in liquid nitrogen for biochemical tests and the other was fixed in 10% formalin and embedded in paraffin for histology. Paraffin blocks were prepared and four‐micrometre serial tissue sections from the liver, spleen and right lung were deparaffinised and stained with haematoxylin and eosin (H&E). Lung sections were also stained with pentachrome, and liver and spleen sections for trichrome and Prussian blue. The entire slide sections were scanned, digitised and analysed using the Aperio ScanScope XT system (Aperio Inc., Vista, CA) and Aperio Image Scope software.

### 2.8 Statistical Analysis

Data were analysed using GraphPad Prism for Windows v.10.4.0 (Boston, MA) and NCSS 2022 statistical software (East Kaysville, UT). The results are presented as mean ± SEM and compared using student's T‐tests for unpaired data and two‐factor ANOVA for four groups with post hoc analyses. Spearman Rho test for non‐parametric data was used for correlations. *p* < 0.05 was considered statistically significant.

## 3 Results

### 3.1 Plasma Levels of PNP Are Markedly Elevated and Correlate With Markers of Hemolysis in Patients With SCD

**FIGURE 1:**
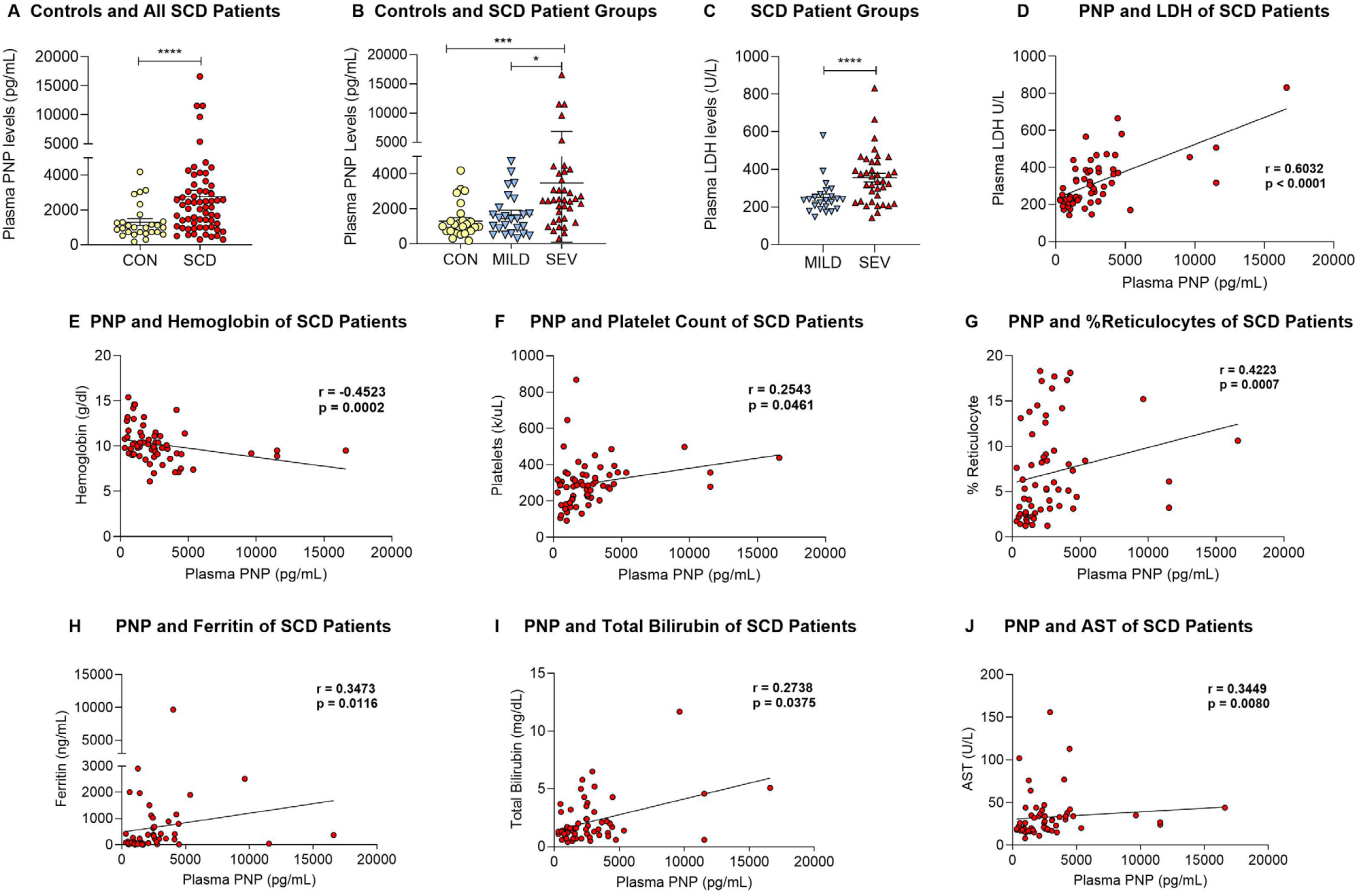
Plasma purine nucleoside phosphorylase (PNP) levels in human controls and adults with SCD and their associations with markers of hemolysis. (A) Plasma PNP levels were markedly elevated in adults with SCD as compared to healthy controls; (B) Adults with severe SCD had significantly elevated plasma PNP levels as compared to those with mild disease and healthy controls; (C) Plasma LDH was higher in the patients with severe SCD than in those with mild SCD; (D) Plasma PNP levels significantly correlated with plasma LDH levels; (E) PNP levels were negatively correlated with haemoglobin; Plasma PNP levels were positively correlated with platelet counts (F), % reticulocytes (G), ferritin (H), total bilirubin (I), and plasma AST (J). Controls (CON): HbAA (*n* = 27), all SCD (*n* = 63) composed of mild and severe (SEV) SCD groups; Mild SCD (*n* = 25): HbSC and HbSB^+^; Severe SCD (*n* = 38): HbSS and HbSB^0^. PNP, Purine Nucleoside Phosphorylase; LDH, Lactate Dehydrogenase; AST, Aspartate Transferase; *p < 0.05, **p < 0.01; ***p < 0.001; Mann–Whitney U test (A and B); Kruskal–Wallis test (C); Spearman Rho correlation test (D—J).

Plasma PNP levels were markedly elevated in the patients compared to controls (SCD: 2772 ± 362 vs. CON: 1310 ± 188.1 pg/mL, *p* = 0.001, Figure 1A) and those with severe disease had higher PNP levels (Severe: 3558 ± 570.9 vs. Mild: 1681 ± 240.9 pg/mL, *p* = 0.003, Figure 1B) and LDH levels (Severe: 373 ± 21.0 U/L vs. Mild: 249.6 ± 14.0 U/L, *p* < 0.0001, Figure 1C) than those with mild disease. The PNP levels in the patients with SCD correlated positively with LDH (*r* = 0.6032, *p* < 0.0001, Figure 1D) and platelet counts (*r* = 0.2543, *p* = 0.0461, Figure 1F), and negatively with haemoglobin (*r* = −0.4523, *p* = 0.0002, Figure 1E). Plasma PNP levels also positively correlated with % reticulocytes (Figure 1G), ferritin (Figure 1H), total bilirubin (Figure 1I) and AST (Figure 1J). As expected, the correlations were generally stronger in the patients with severe disease (Table S3).

### 3.2 Townes AA and SS Mice Efficiently Convert 8‐AG to 8‐Aminoguanine

**FIGURE 2:**
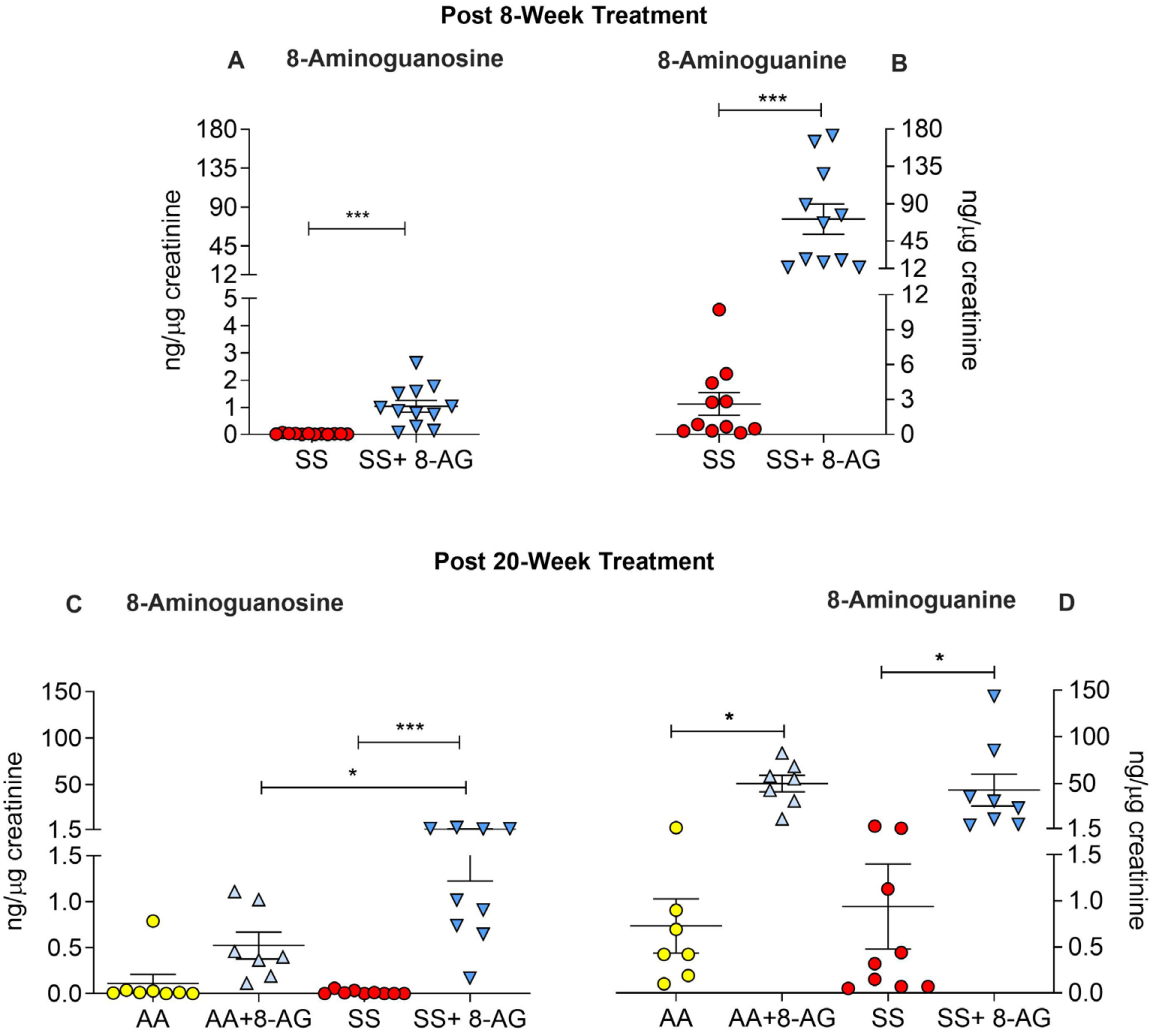
Townes AA and SS mice efficiently convert 8‐aminoguanosine (8‐AG) to 8‐aminoguanine. (A) After 8 weeks of treatment, untreated Townes SS mice had non‐detectable levels of urinary 8‐AG, while the 8‐AG‐treated SS mice had very low urinary concentration of 8‐AG. (B) Eight weeks of treatment with 8‐AG yielded a 40–50‐fold increase of urinary 8‐aminoguanine (the active PNP inhibitor form) in the treated SS mice, while levels of the endogenous 8‐aminoguanine remained low in the untreated SS mice. (C) Long‐term (20 weeks) treatment with 8‐AG yielded only a small increase of urinary 8‐AG in SS mice; and (D) a 40‐fold increase of the active inhibitor, 8‐aminoguanine, in both treated Townes AA and SS. Townes SS mice (SS), Townes AA mice (AA), and Townes SS mice with 8‐AG treatment (SS + 8AG), Townes AA mice with 8‐AG treatment (AA+8AG). *p < 0.05, ***p < 0.001, unpaired *t*‐test (A and B), one‐way ANOVA (C and D), *n* = 7–12 per group.

First, we examined whether Townes mice efficiently metabolise 8‐AG (a pro‐drug) to 8‐aminoguanine, an endogenous purine metabolite and a potent PNP inhibitor [47]. After 8 and 20 weeks of 8‐AG treatment (Figure 2, top and bottom panel, respectively), the SS mice had very negligible urinary concentrations of 8‐AG, albeit higher in the treated SS mice than the untreated group, *p* < 0.001 (Figure 2A,C). The SS mice treated with 8‐AG for 8 or 20 weeks had 40–50‐fold higher urinary concentration of the active PNP inhibitor 8‐aminoguanine, while the urinary levels of 8‐aminoguanine remained low in the untreated SS mice, *p* < 0.001 (Figure 2B,D). This indicates efficient conversion of the pro‐drug and weak PNP inhibitor 8‐AG to the potent PNP inhibitor 8‐aminoguanine in 30‐week‐old Townes SS mice even at an advanced stage of disease (Figure 2C,D). Notably, in *naïve* mice (animals not receiving oral 8‐AG), no endogenous 8‐AG was measurable, whereas endogenous 8‐aminoguanine was detectable in the urine, confirming our previous findings that 8‐aminoguanine is an endogenous metabolite [48].

### 3.3 8‐AG Inhibits PNP Activity, Rebalances Dysregulated Purine Metabolism, and Has No Adverse Haematological Effects in SS MICE

**FIGURE 3:**
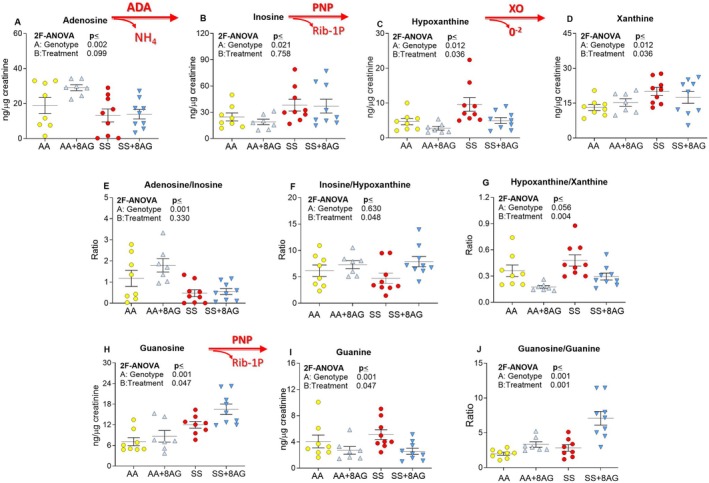
Purine urinary metabolome in Townes AA and SS mice. Urinary adenosine (A) was lower in control SS mice compared with the untreated AA mice and 20‐week treatment with 8‐AG did not affect urinary adenosine levels. Urinary inosine (B), the catabolic product of ADA activity, was higher and the adenosine/inosine ratio (E) was lower in untreated SS mice compared with untreated AA mice suggesting increased ADA activity in SS mice; 8‐AG treatment did not affect urinary adenosine or inosine levels confirming that 8‐AG does not inhibit ADA. The urinary levels of the direct products of PNP activity, hypoxanthine (C) and guanine (I) were higher in the untreated SS mice than in AA controls, and notably, 8‐AG treatment reduced these levels, increased inosine/hypoxanthine ratio (F), guanosine (H) levels and guanosine/guanine ratio (J). This suggests efficient PNP inhibition by 8‐AG. Furthermore, urinary xanthine levels (D) and the hypoxanthine‐xanthine ratio (G) were reduced in the 8‐AG‐treated SS mice suggesting a reduction in XO activity. This indicates an indirect effect of 8‐AG due to reduced hypoxanthine and subsequently reduced downstream production of xanthine. Arrows indicate the direction of the metabolic reaction. Purine nucleoside phosphorylase (PNP), adenosine deaminase (ADA), ribose‐1‐phosphate (Rib‐1P); Townes SS mice (SS), Townes AA mice (AA), Townes SS mice with 20 weeks of 8‐AG (SS + 8AG), Townes AA mice with 20 weeks of 8‐AG (AA+8AG), two factor ANOVA (2F‐ANOVA); *n* = 7–12 per group.

Analysis of the urinary purine metabolome revealed dysregulated purine metabolism in SS mice compared to age‐matched AA controls (Figure 3). The 30‐week‐old SS mice had reduced urinary adenosine (genotype effect *p* = 0.002) and lower adenosine/inosine ratio (*p* = 0.001; Figure 3A,E, respectively) and increased inosine (*p* = 0.021), hypoxanthine (*p* = 0.012) and xanthine levels (*p* = 0.017; Figure 3B–D, respectively), collectively suggesting accelerated purine metabolism and increased activity of ADA, PNP and XO [13, 14, 15]. The higher urinary hypoxanthine levels along with increased guanine levels (*p* = 0.001, Figure 3I) are also indicative of increased PNP activity as previously reported in SCD patients [13] and in accordance with the elevated plasma PNP levels in patients from the current study (Figure 1). The 20‐week treatment with 8‐AG reduced hypoxanthine (*p* = 0.036, Figure 3C) and guanine (*p* = 0.047, Figure 3I) and increased the inosine/hypoxanthine ratio (*p* = 0.048, Figure 3F) and guanosine/guanine ratio (*p* = 0.001, Figure 3J), suggesting an efficient inhibition of PNP by 8‐AG in SCD mice (see Figure S1C,G,I,J). Furthermore, 8‐AG treatment did not alter xanthine levels in the treated SS mice (Figure S1D), suggesting no effects of PNP inhibition on XO activity, but reduced the hypoxanthine/xanthine ratio (*p* = 0.004, Figure 3G), most likely as an indirect effect of 8‐AG due to reduced hypoxanthine and subsequently reduced downstream production of xanthine.

**FIGURE 4:**
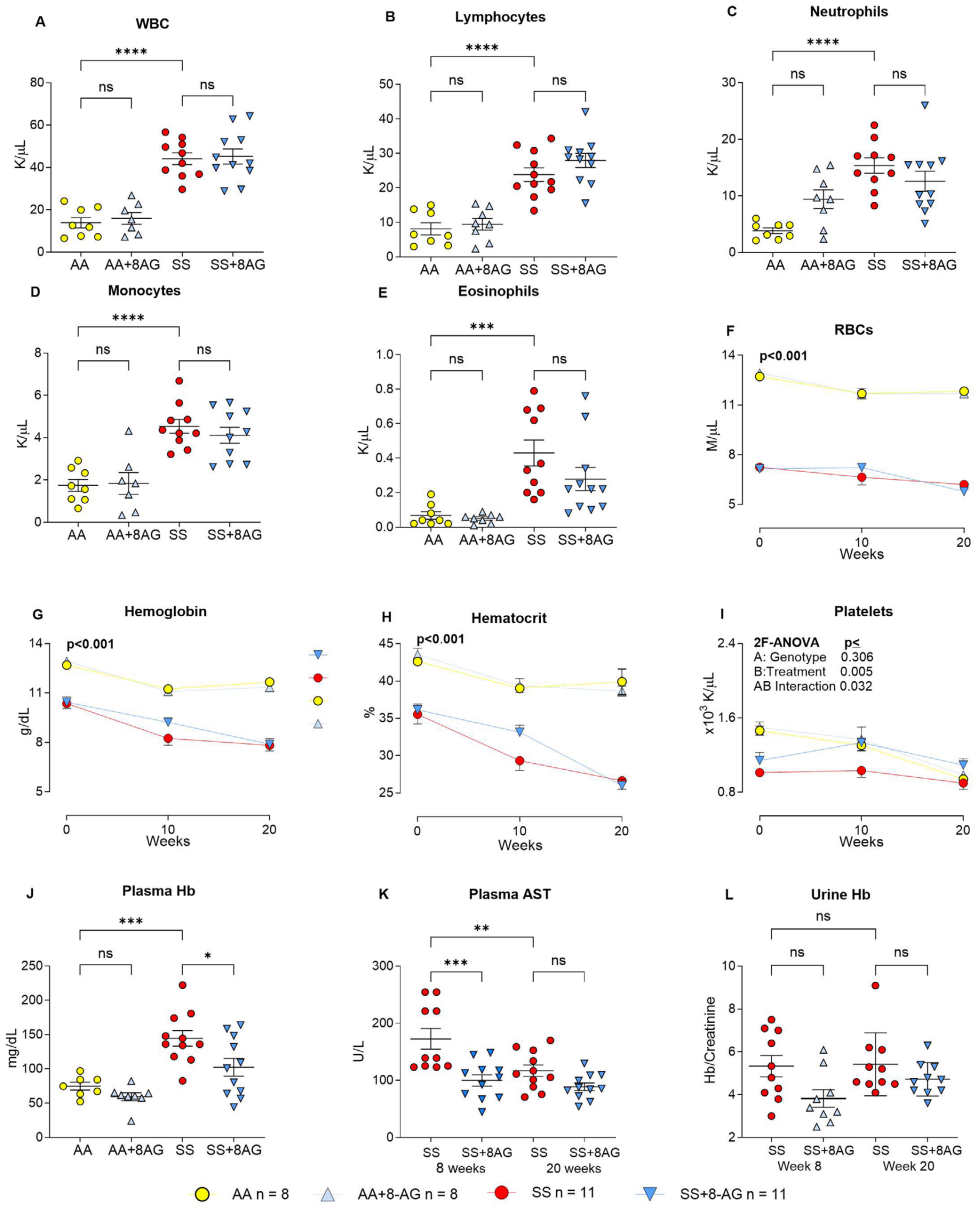
Haematological parameters and markers of hemolysis in Townes SS and AA mice treated with or without 8‐AG. (A–E): Total white blood cell counts and differentials in Townes AA and SS mice treated with or without 8‐AG for 20 weeks. The untreated SS mice had higher total WBC (A), absolute lymphocyte (B), absolute neutrophil (C), absolute monocyte (D) and absolute eosinophil (E) counts than the untreated AA mice. 8‐AG treatment had no adverse effect on the circulating levels of these immune cells. (F–L):RBC parameters, platelet count and markers of hemolysis in Townes AA and SS mice treated with or without 8‐AG. After 20 weeks, the untreated mice had declining RBC (F), haemoglobin (G) and haematocrit (H) compared to the untreated AA mice. After the initial 10 weeks of treatment, 8‐AG slowed down the anaemia progression, but this effect was lost after 20 weeks. (I): Townes SS mice had lower platelet counts that were increased by 8‐AG treatment . (J): Untreated SS mice had higher free plasma haemoglobin than the AA mice, and 8‐AG treatment reduced plasma haemoglobin in SS mice; (K, L): Effect of short‐ (8 weeks) and long‐term (20 weeks) treatments with 8‐AG on the markers of hemolysis in SS mice. 8‐AG treatment tended to reduce plasma AST levels (K) and hemoglobinuria (L) in the SS mice at both 8 and 20 weeks. Townes SS mice (SS), Townes AA mice (AA), Townes SS mice with 8‐AG (SS + 8AG), Townes AA mice with 8‐AG (AA+8AG). White blood cells (WBC), red blood cells (RBC), *p < 0.05, ***p < 0.001, ****p < 0.0001, not significant (NS), (A–E) One‐way ANOVA; (F–L) 2 factor ANOVA (2F‐ANOVA), *n* = 8–11 per group.

Complete deficiency of PNP (a very rare, inherited disorder) leads to severe combined immunodeficiency, lymphopenia, autoimmune hemolytic anaemia and thrombocytopenia in humans [49]. Thus, we monitored mice blood counts at 0, 10, and 20 weeks of treatment (Figure 4 and Figure S2). As expected, compared to untreated AA mice, the untreated SS mice had higher total white blood cell (WBC, Figure 4A), lymphocyte (Figure 4B), neutrophil (Figure 4C), monocyte (Figure 4D), and eosinophil (Figure 4E) counts. Notably, 8‐AG treatment did not significantly reduce the circulating levels of immune cells in both AA and SS mice.

### 3.4 8‐AG Reduces Hemolysis and Increases Platelet Counts in SS Mice

To further investigate the impact of 8‐AG treatment, we analysed serial RBC parameters in both Townes AA and SS mice during the 20‐week treatment with 8‐AG versus control animals consuming tap water (*n* = 9–11 per group). As expected, because of chronic hemolysis, the SS mice were anaemic throughout the study period compared to the AA mice (Figure 4F–H and Figure S2). However, whereas the untreated SS mice had worsening anaemia, the progression of anaemia halted at around the 10th week of treatment in the 8‐AG‐treated SS mice suggesting reduced hemolysis. To confirm reduction of hemolysis with 8‐AG treatment, we analysed mouse plasma samples for free haemoglobin and AST and urine for free haemoglobin levels (Figure 4J–L). Plasma free haemoglobin was reduced following 20 weeks of 8‐AG treatment in the SS mice (Figure 4J). Levels of both plasma AST and urinary haemoglobin in the SS mice treated for 8 and 20 weeks were lower than in untreated SS mice (Figure 4K,L), however, the results did not reach statistical significance.

Compared to AA controls, SS *naïve* mice had reduced platelet counts. 8‐AG had no effect on platelet counts in AA mice, but importantly led to a transient increase in platelet counts in SS mice at 10 weeks (treatment *p* = 0.005; time ×treatment interaction *p* = 0.032; Figure 4I).

### 3.5 8‐AG Improves Right Ventricular (RV) Function, Reduces RV Hypertrophy and Does Not Impact Left Ventricular (LV) Function and Structure

**FIGURE 5:**
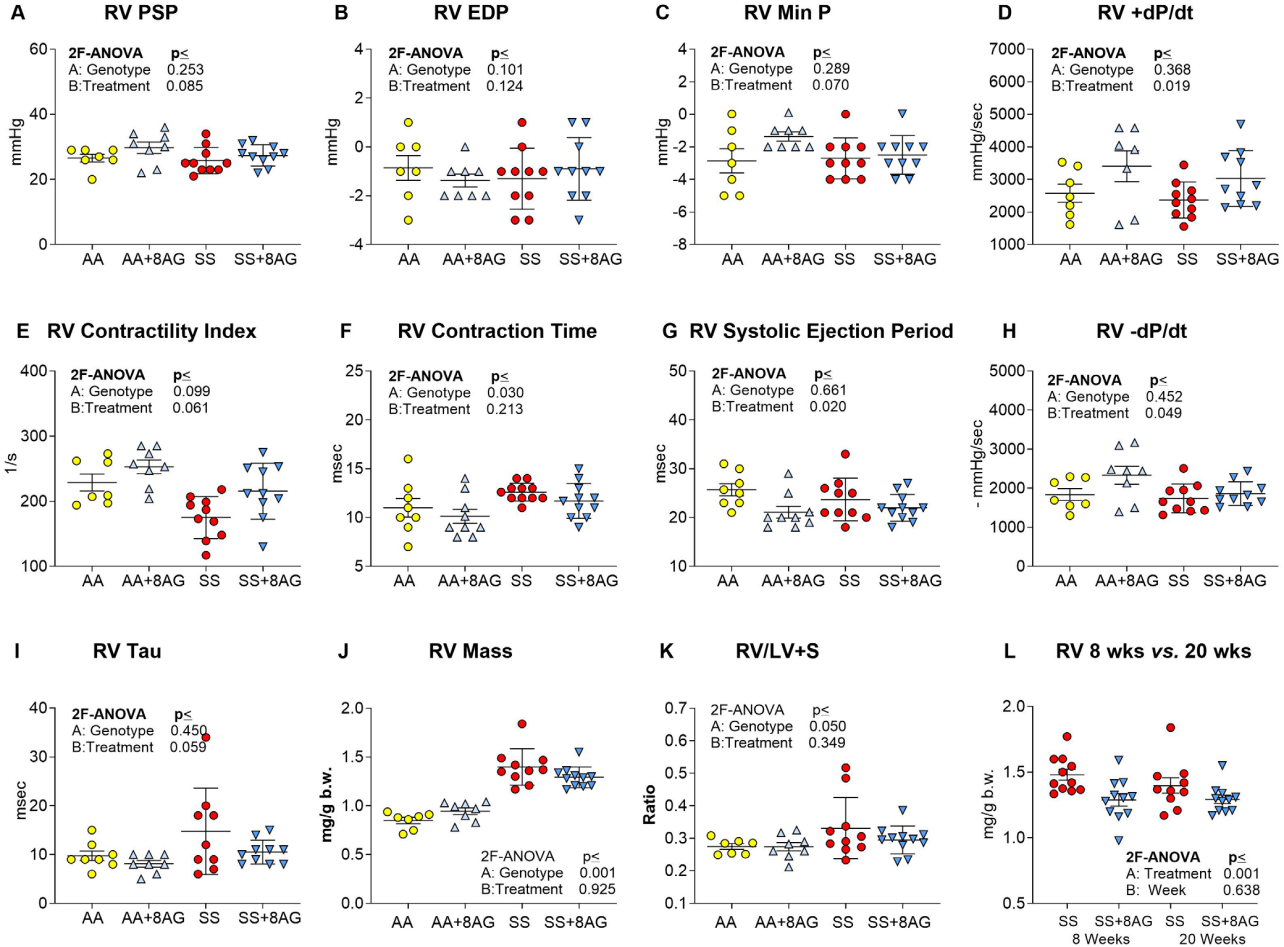
Right ventricular pressures and function in Townes AA and SS mice measured by closed‐chest catheterization. Following 20 weeks of treatment with or without 8‐AG, both Townes AA and SS mice underwent closed‐chest RV catheterization. (A–C): RV peak systolic pressure, RV end‐diastolic pressure and RV Min P were not different among groups; (D) 8‐AG significantly increased the RV pressure rate of rise (+dP/dt) in both AA and SS mice; (E) The genotype had no effect on the RV contractility index, but treatment tended to improve contractility; (F) The RV contraction time was higher in SS mice compared to AA controls; (G) The RV systolic ejection period was reduced significantly with 8‐AG treatment; (H) 8‐AG significantly increased the RV pressure rate of decline (−dP/dt) in both AA and SS mice; (I) RV tau, a marker of relaxation, was not affected by genotype but showed a trend towards reduction with 8‐AG treatment; SS mice had RV hypertrophy (J) with increased Fulton Index (RV/LV + S) (K); There was remarkable reduction of the RV hypertrophy in the SS mice following short (8 weeks) and long (20 weeks) term treatments with 8‐AG (L). Townes SS mice (SS), Townes AA mice (AA), Townes SS mice with 8 or 20 weeks of 8‐AG (SS + 8AG), Townes AA mice with 8 or 20 weeks of 8‐AG (AA+8AG), 2‐ factor ANOVA (2F‐ANOVA). (*n* = 7–10 per group).

Humans with SCD have high rates of hemolysis and are at risk of pulmonary hypertension (PH) [50]. SCD mice also develop PH and RV dysfunction [51]. Thus, we investigated the effect of 8‐AG treatment on RV hemodynamics, measured by closed‐chest RV catheterization (Figure 5A–L), and on echocardiographic parameters of LV dysfunction (Figure S4) in subsets of Townes AA and SS mice treated with 8‐AG and control animals (*n* = 7–10 per group). Compared to AA controls, non‐treated SS mice did exhibit RV dysfunction in terms of contractile function (Figure 5E,F) and RV hypertrophy (Figure 5J,K). Following 20‐week treatment, 8‐AG tended to improve RV contractility in both AA and SS mice as evidenced by increased RV pressure rate of rise (dP/dt; *p* = 0.019; Figure 5D), a tendency towards increased contractility index (*p* = 0.061; Figure 5E), and a reduction in the systolic ejection period (*p* = 0.020; Figure 5G). In both AA and SS mice, 8‐AG improved RV relaxation as evidenced by increased −dP/dt (*p* = 0.049, Figure 5H) and a tendency to reduce RV Tau in 8‐AG treated animals (*p* = 0.059; Figure 5I). Most importantly, both short‐ and long‐term treatments with 8‐AG reduced RV hypertrophy (Figure 5L). Histopathological analysis of lung tissue from control and treated AA and SS mice revealed mild to moderate pathology with scattered mononuclear infiltrates, emphysema, perivascular edema and dilated and congested/clogged small‐size pulmonary arteries, and no signs of haemorrhages and infarcts in untreated SS mice (Figure S3). 8‐AG treatment did not modify these mild/moderate histopathological alterations.

Non‐invasive echocardiography revealed LV hypertrophy and dilated cardiomyopathy with preserved ejection fraction in SS mice compared to AA controls, and 20‐week treatment with 8‐AG did not affect LV function (Figure S4).

### 3.6 8‐AG Attenuates Kidney Injury in SS Mice

**FIGURE 6:**
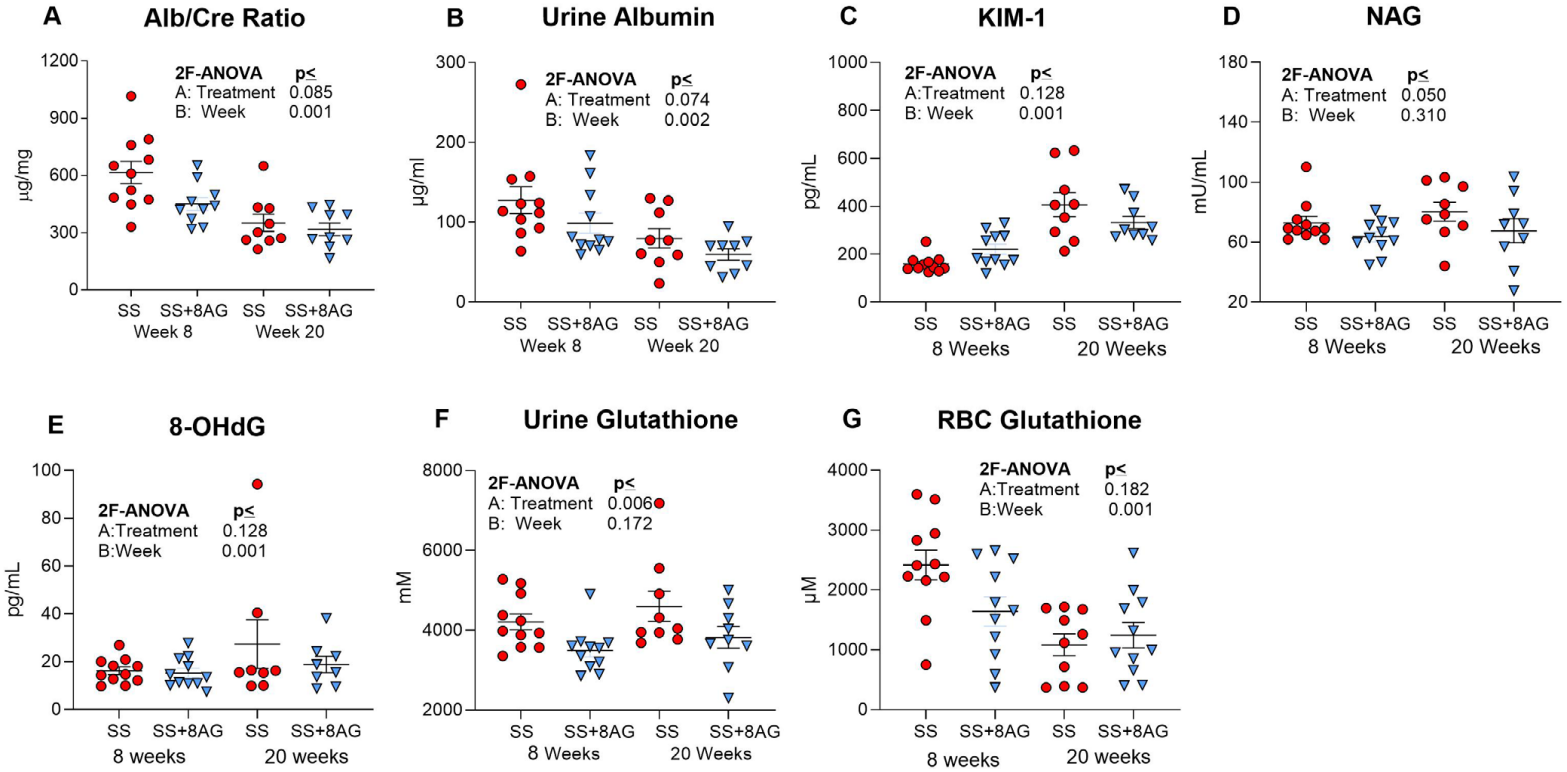
Markers of renal damage in Townes SS mice treated with or without 8‐AG. In SS mice, 8‐AG tended to reduce albuminuria (A, B) and urinary KIM‐1 (C); 8‐AG treatment significantly attenuated urinary NAG activity (D) and glutathione urinary excretion (F). There was a time‐related reduction in erythrocytic glutathione in (G). The changes in markers of renal damage are suggestive of both reduced tubular and glomerular injury by 8‐AG in SS mice. Townes SS mice (SS), Townes SS mice with 8 or 20 weeks of 8‐AG (SS + 8AG), 2 factor ANOVA (2F‐ANOVA). (*n* = 7–10 per group).

SCD‐related kidney disease occurs in 50% of patients, is an independent risk factor for premature death [52, 53], and is characterised by both glomerular and tubular damage [54]. In SS mice, 8‐AG significantly attenuated urinary N‐acetyl‐beta‐D‐glucosaminidase (NAG) activity (Figure 6D) and glutathione urinary excretion (Figure 6F) and, to a lesser degree, urinary kidney injury molecute‐1 (KIM‐1, Figure 6C), suggesting reduced tubular damage, and decreased albuminuria (Figure 6A,B), which is indicative of reduced glomerular damage. In SS mice, the progression of disease was associated with increased 8‐hydroxydeoxyguanosine (8‐OHdG) levels (Time: *p* = 0.001, Figure 6E), a marker of oxidative stress, and a time‐related reduction in erythrocytic glutathione, which should be expected to contribute to increased oxidative stress (Time: *p* = 0.001, Figure 6G). However, treatment with 8‐AG did not affect levels of 8‐OHdG or erythrocytic glutathione.

### 3.7 8‐AG Reduces Hepatomegaly, Liver Injury and Splenomegaly in SS Mice

**FIGURE 7:**
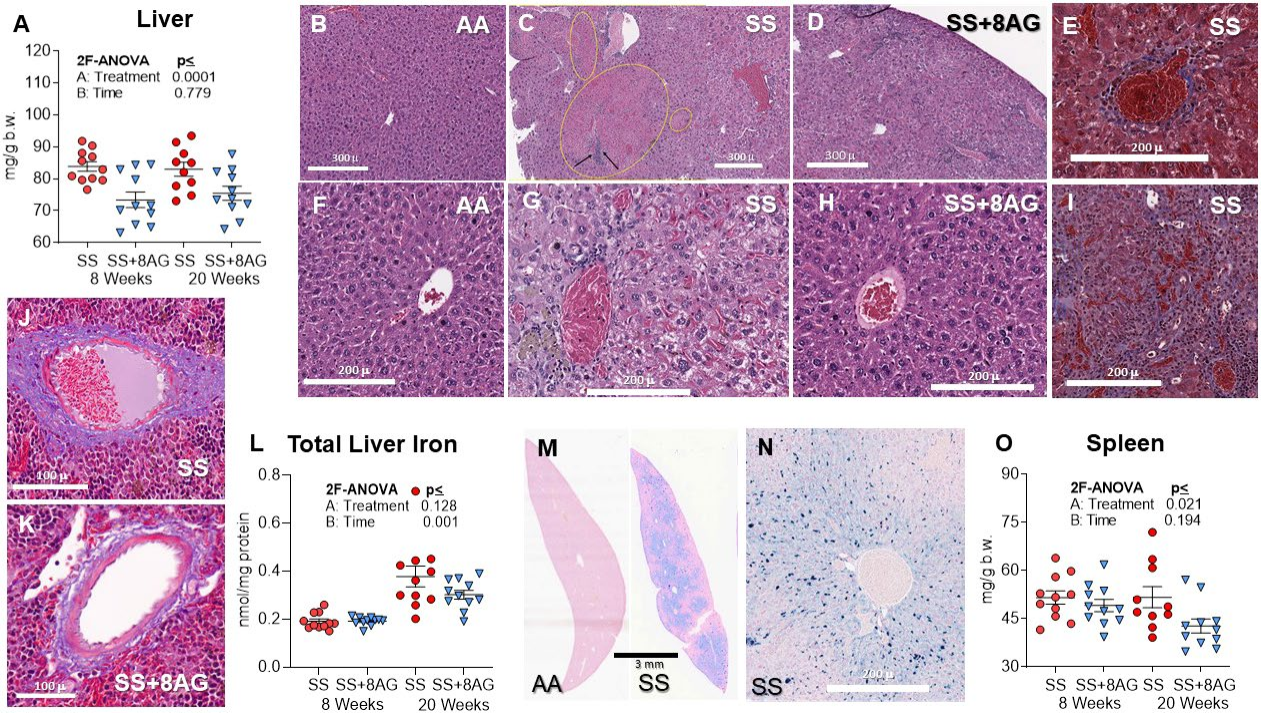
Hepatomegaly, liver injury and splenomegaly in Townes AA and SS mice treated with or without 8‐AG. The SS mice had hepatomegaly compared to the AA mice and the 8‐AG treatment reduced the liver weight (A). Compared to AA controls (B, F), SS mice exhibited ischemic and necrotic areas with infiltration by inflammatory cells (oval circles and black arrows for inflammatory cells), (C) and hepatocellular damage (G) and pericellular, perisinusoidal and perivascular fibrosis (E, I, J); 8‐AG treatment tended to attenuate ischemic/necrotic injury (D), hepatocellular damage (H) and perivascular fibrosis (K). Remarkable iron deposition was seen in SS mice in central and mid hepatic areas (M), mainly around central veins and nearby sinusoids (N) and 8‐AG tended to reduce iron deposition (L). Both 8‐ and 20‐week 8‐AG treatment reduced splenomegaly (O). (B–D and F–H) Haematoxylin and eosin; (E, I–K) Trichrome; M and N: Prussian blue staining. Scale bars: 300 μm (B–D, N), 60 μm (J, K) and 200 μm (E–I). Townes SS mice (SS), Townes SS mice treated with 20 weeks of 8‐AG (SS + 8AG), 2 factor ANOVA (2F‐ANOVA). (*n* = 7–10 per group).

In patients with SCD, the prevalence of liver disease ranges from 10% to 30% at autopsy and is associated with increased mortality [55, 56]. As expected, compared to the 30‐week‐old Townes AA controls, the untreated SS mice had remarkably larger livers (Liver: AA = 2230 ± 73 vs. SS = 2886 ± 81 mg, *p* = 0.001; Liver Index: AA = 57 ± 1 vs. SS = 83 ± 2 mg/g b.w. *p* < 0.001). Compared to control AA mice (Figure 7B,F), SS mice had ischemic and necrotic areas with infiltrating inflammatory cells (Figure 7C). The central veins and sinusoids were congested with sickled RBCs (Figure 7E,G,I) and surrounded by inflammatory cells and pericellular (Figures 7E,I) and perivascular (Figure 7J) fibrotic changes. Furthermore, SS mice exhibited remarkable iron deposition in central and mid‐hepatic areas (Figure 7M), mainly around central veins and nearby sinusoids (Figure 7N). Both 8‐ and 20‐week treatment with 8‐AG significantly reduced hepatomegaly (treatment effect, *p* = 0.001 Figure 7A), tended to reduce ischemic/necrotic damage (Figure 7D), central veins and sinusoids congestion and liver iron content (Figure 7H,L, respectively) and perivascular fibrosis (Figure 7K). Townes SS mice had massive splenomegaly (Spleen AA = 165 ± 13 vs. SS = 1813 ± 144 mg; *p* < 001; Spleen index: AA = 4 ± 0.3 vs. SS = 51 ± 32 mg/g b.w., *p* = 0.0001). Histopathological changes included vascular congestion and loss of the splenic architecture in SS mice, and iron deposition in both AA and SS mice (Figure S5). Notably, both 8‐ and 20‐week 8‐AG treatment reduced splenomegaly in SS mice (*p* = 0.021, Figure 7O).

## 4 Discussion

PNP is a key enzyme in purine metabolism, and purine metabolism plays an important role in RBC physiology and pathology [11, 57, 58], yet its role in SCD and other hemolytic diseases is unknown. The notion that, per cell volume, RBCs have the highest level of PNP [12, 59], suggests that under hemolytic conditions PNP may be an unrecognised important eDAMP. Here, we show for the first time that in adults with SCD, PNP levels are higher than in their age‐ and race‐matched HbAA controls; these levels are associated negatively with anaemia, and positively with LDH and other hemolytic parameters. Similarly, we demonstrate that PNP activity is higher in humanised SS mice compared to AA controls and show that, in SS mice, 8‐AG attenuates hemolysis. Notably, 8‐AG improves SS RBC deformability and sickling, which may explain reduced hemolysis in Townes SS mice [60]. These findings are consistent with our initial report of increased PNP activity and accelerated purine metabolism in paediatric SCD patients and animals exposed to a hemolytic insult [12, 13, 14, 15]. PNP is a ubiquitous and largely intracellular enzyme that is present not only in RBCs but also in neighbouring endothelial cells and platelets, three key cells involved in the complex pathobiology of SCD. Thus, PNP may be implicated in multiple pathologies that are relevant to SCD, including RBC sickling, hemolysis, hemolytic angioproliferative injury and cell aggregation [39]. These considerations motivated us to test the hypothesis that PNP inhibition may be beneficial in SCD.

The increased erythrocytic PNP release into the bloodstream and extravascular compartment may have two important consequences pertinent to the complex pathophysiology of SCD [15]. First, the accelerated guanosine/inosine metabolism by abundantly present extracellular PNP would reduce the antioxidative [23, 24, 25, 26], anti‐ischemic [27, 28, 29, 30], anti‐inflammatory [24, 31, 32], antiplatelet/antithrombotic [33, 34] and anti‐nociceptive effects [35, 36, 37, 38] of guanosine/inosine, which are all highly desirable in SCD. Second, increased PNP activity (largely on its own, but also in concert with ADA and GDA) would result in overproduction and accumulation of vasculotoxic hypoxanthine and xanthine [22] that cause oxidative stress‐induced endothelial dysfunction [19, 22], have pro‐inflammatory effects, and are involved in blood cell aggregation, ischemia/reperfusion injury [61] and chronic pain. This would further exacerbate the existing inflammatory and oxidative injury and thereby worsen organ damage in SCD. Our study found that inhibiting PNP with 8‐AG reduced the production of xanthine and hypoxanthine, the vasculotoxic substrates of XO. Thus, 8‐AG has an upstream target in the purine metabolic pathway that could efficiently prevent the production of xanthines. Notably, elevated intravascular XO causes vasculotoxicity in hemolytic anemia, and its inhibition by febuxostat—an XO inhibitor and anti‐gout drug—reduces hemolysis and vascular injury in SCD mice [62].

This study confirms that 8‐AG is an endogenous purine metabolite that is rapidly converted to 8‐aminoguanine (an active PNP inhibitor) and demonstrates that in 30‐week‐old SS mice at an advanced stage of disease, conversion of 8‐AG to 8‐aminoguanine is not altered. The increased urinary inosine and guanosine levels in 8‐AG treated SS mice are in agreement with a previous report of a 3–4 fold increase in the half‐life of inosine and guanosine by 8‐AG [63], which would be expected to increase their SCD‐protective effects. Indeed, in this study, long‐term oral treatment of SS mice with 8‐AG not only elevated inosine and guanosine levels but also reduced the downstream production of the vasculotoxic hypoxanthine and xanthine [19]. This rebalancing of the purine metabolome resulted in reduced end‐organ damage. Treatment of SS mice with 8‐AG reduced hemolysis as evidenced by reduced plasma Hb, AST and hemoglobinuria. Also, 8‐AG treatment attenuated hepatomegaly, liver damage, splenomegaly and kidney damage, reduced RV hypertrophy and tended to improve RV systolic/diastolic function in Townes SS mice.

Another noteworthy finding is that in SS mice, long‐term treatment with 8‐AG led to a transient increase in the platelet count (Figure 4I). This effect was most likely attributable to reduced splenic sequestration of platelets splenomegaly and hypersplenism improve with decreased hemolysis, and possibly from reduced platelet aggregation with other blood cells that accompanies vaso‐occlusion during sickle cell crises. This deserves further investigation.

A complete lack of PNP activity results in severe immunodeficiency syndrome (SIDS) [64] but it is important to note that heterozygotic parents of PNP‐deficient patients with only 20%–30% of normal PNP activity are healthy [65]; and, as shown previously [66], only 8%–11% PNP activity is required for normal neurological and immune function. Thus, it is rational to predict that partial inhibition with a moderately potent PNP inhibitor such as 8‐AG would be safe in SCD. Indeed, long‐term inhibition of PNP in this study did not cause adverse haematological effects. This is in agreement with recent long‐term studies in several rat disease models treated with 8‐AG or 8‐aminoguanine for up to 20 weeks, showing the safety of partial PNP inhibition [48, 67, 68, 69].

The findings that the levels and metabolic activity of PNP are markedly enhanced in SCD and correlate well with hemolysis suggest that PNP should be considered as a promising therapeutic target in SCD. In this regard, monitoring PNP activity and purine metabolite levels could serve as a novel biomarker of therapeutic response and offer a precision medicine approach in SCD. Monitoring PNP levels could be useful to identify SCD patients who would benefit most from the treatment and tailor the posology of a PNP inhibitor to PNP levels and/or activity, as well as the purine plasma levels of the patient [39].

Our study has several limitations. While Townes mice are an excellent preclinical SCD model, the beneficial effect of 8‐AG on splenomegaly would not be expected in patients with the severe subtypes of SCD as these individuals usually undergo autosplenectomy before adulthood due to recurrent splenic infarctions. In addition, when extrapolating data from SCD animals to humans, it should be noted that human RBCs are the richest source of PNP, whereas considerably lower erythrocytic PNP levels are present in mice and rats [12, 70]. Whether Townes SS mice have lower or similar erythrocytic PNP levels to patients with SCD needs to be investigated.

In conclusion, we found that inhibition of PNP by 8‐AG has multipronged beneficial effects in SCD. Our findings should prompt further investigation of the mechanisms and beneficial effects of PNP inhibition in SCD.

## Author Contributions

L.L.‐I. performed experiments, analysed the data and edited the manuscript, S.M.M. performed experiments, E.K.J. interpreted the data and critically reviewed the manuscript, E.M.N. and S.P.T. developed the ideas, designed the study, interpreted the data, and edited the manuscript.

## Funding

This study was supported in part by an award from The Precision Medicine Institute in partnership with PittSciVelo from the University of Pittsburgh (SPT, EMN, EKJ), P3HVB award in Haemostasis and Vascular Biology, funded by the University of Pittsburgh Vascular Medicine Institute, Vitalant and The Hemophilia Center of Western Pennsylvania (SPT, EMN) and an American Society of Hematology Inclusion Pathway (HIP) Fellow Award (AEA).

## Ethics Statement

We obtained deidentified banked citrated plasma samples and laboratory parameters collected from patients with SCD (*n* = 63) during steady state as well as race, age, and sex‐matched HbAA controls (*n* = 27) enrolled in a prior clinical trial (NCT02946905).

## Consent

All participants gave consent.

## Conflicts of Interest

E.K.J. and S.P.T. are co‐inventors on US # 11,103,526, B2 patent covering the use of PNP inhibitors in SCD, angioproliferative vasculopathy, and pulmonary hypertension. E.M.N. is an advisory board/consultant for Novo Nordisk, Novartis and Chiesi.

## Supporting information


**Appendix S1:** jcmm70996‐sup‐0001‐AppendixS1.docx.

## Data Availability

The data that support the findings of this study are available from the corresponding author upon reasonable request.
