# Supplementary material for: Purine Nucleoside Phosphorylase Inhibition Rebalances Purine Metabolism and Attenuates Organ Damage in Sickle Cell Mice
Source: J Cell Mol Med. 2025 Dec 30;29(24):e70996. doi: 10.1111/jcmm.70996 (PMC12753582; doi:10.1111/jcmm.70996)
Supplement: Supplementary file 1 — Appendix S1: jcmm70996‐sup‐0001‐AppendixS1.docx. [file JCMM-29-e70996-s001.docx]

**Purine Nucleoside Phosphorylase Inhibition Rebalances Purine Metabolism and Attenuates Organ Damage in Sickle Cell Mice**

**Running title:** PNP Inhibition Attenuates Organ Damage in SCD Mice

Adekunle Emmanuel Alagbe^1,3^, Lynda Little-Ihrig^1,3^, Stephanie M. Mutchler^3^, Edwin K. Jackson^2^, Enrico M. Novelli^1,3*^, Stevan P. Tofovic^1,2,3*^

Heart, Lung, Blood and Vascular Medicine Institute^1^ and Department of Pharmacology and Chemical Biology^2^ and Medicine^3^, University of Pittsburgh School of Medicine, Pittsburgh, Pennsylvania, USA

**Supplemental Materials**

**Methods**

***Non-invasive transthoracic echocardiography***

Non-invasive transthoracic echocardiography was performed by the University of Pittsburgh Small Animal Ultrasonography Core using the Visualsonics Vevo 3100 ultrasound machine (FUJIFILM VisualSonics, Toronto, Canada) as previously described.^1,2^ The mouse was anesthetized with 3% isoflurane and gently placed in supine position on a warming pad maintained at 37^o^C. A toe pinch was performed to confirm the sedation and the heart rate was maintained at 400 - 500 bpm. The mouse was gently restrained by taping the limbs into the ECG leads with electrode gel. Depilation cream for hair removal was applied to the chest and upper abdomen of the mouse and eye lubricant was applied to the eyes to prevent drying of the sclera. Ultrasound gel was applied to the chest and upper abdomen and the MX400 transducer was gently placed on the chest. Images were acquired on the VisualSonics Vevo 3100 ultrasound machine within 15-20 minutes per mouse. Two-dimensional measurements were taken from the left ventricular short axis at the papillary muscle (M-Mode). Apical 4-chamber view images were taken at the mitral valve inflow level.

The images were analyzed by a blinded sonographer using the Vevo Lab 5.5.1, Visual Sonics imaging software. Means of triplicate beats were obtained for LV ejection fraction (LVEF), end-diastolic volume (EDV), end-systolic volume (ESV), LV internal diameter in diastole (LVIDd) and in systole (LVIDs), LV posterior wall thickness in diastole (LVPWd), and systole (LVPWs), interventricular septum thickness in systole (IVs), heart rate (HR), cardiac output (CO), and LV fractional shortening (LVFS). These data are presented after normalizing to animal weight. After the procedure, the transducer and the tape were removed from the limbs.The isoflurane was discontinued and the mouse was allowed to recover. The mouse was then returned to the cage for monitoring until full recovery.

***Closed-chest right heart micro-catheterization and in vivo RV pressure measurement***

Closed-chest right heart micro-catheterization with *in vivo* RV pressure measurement was performed by the University of Pittsburgh Small Animal Hemodynamic Core as previously described.^3,4^

The mouse was sedated with 1.5-3.0% isoflurane, anesthetized with injected doses of urethane and etomidate, placed on a warm pad maintained at 37^o^C, and tracheostomy intubation was done. The mouse was mechanically ventilated throughout the hemodynamic studies. Closed-chest catherization using a micro pressure–volume Millar catheter was initiated percutaneously. The neck area was cut down to expose the main jugular vein which was isolated and tied off anteriorly. A small incision was made in the vein and the catheter inserted. A loose suture was placed around the vein/catheter to prevent blood loss and the catheter was introduced into the right ventricle for the continuous measurement of the cardiac hemodynamic parameters. Hemodynamic parameters, including all pressures and tau levels and contractility index (CI=dPdtmax/mPAP), were obtained and calculated/analyzed using the IOX2 Software (EMKA Paris, France). Immediately afterwards, the mouse was euthanized, the right and left ventricles as well as the septum was harvested and weighed, and the values used for the calculation of the Fulton Index (RV vs LV+septum ratio). The harvested tissue samples were processed as either snap-frozen or fixed for histology.

**Table s1: List of reagents**

| **Assay** | **Reagent/Source/ Cat No** |
| --- | --- |
| Urine and serum creatinine levels | Creatinine (Enzymatic) Reagent  SetPointe Scientific C7548-120 |
| Urine albumin | Mouse Albumin ELISA Kit  Bethyl Laboratories E99-134 |
| Hemoglobin plasma and urine | QuantiChrom™ Hemoglobin Assay Kit BioAssay Systems DIHB-250 |
| Plasma AST | AST Activity Assay Kit  Sigma-Aldrich MAK055 (This kit is no longer available)  EnzyChrom™ Aspartate Transaminase Assay Kit BioAssay Systems EASTR-100 |
| Mouse PNP ELISA | Mouse Purine Nucleoside Phosphorylase (PNP) ELISA Kit  My BioSource MBS2602982 |
| Human PNP ELISA | Human Purine Nucleoside Phosphorylase (PNP) ELISA Kit, Abcam ab260069 |
| Mouse N-acetylglucosaminidase (beta-NAG) Activity (colorimetric) | N-acetylglucosaminidase (beta-NAG) Activity Assay Kit (colorimetric)  Abcam ab204705 |
| Mouse KIM-1 ELISA | Mouse KIM-1 ELISA Kit (TIM-1) Abcam ab119596 |
| 8-OH 2dG ELISA | 8-hydroxy 2 deoxyguanosine ELISA Kit  Abcam ab201734 |

**Results**

***Laboratory parameters of controls and SCD patients.***

There were 27 HbAA controls and 63 patients with SCD [38 patients with severe disease (HbSS and HbSB0) and 25 with mild disease (HbSC and HbSB+)]. A summary of the laboratory parameters of the study participants is in Table s2.

**Table s2: Laboratory parameters of controls and SCD participants**

| Parameters | Controls  (a) | Mild disease (b) | Severe disease (c) | p-value |
| --- | --- | --- | --- | --- |
| Phenotypes | HbAA  n = 27 | HbSC, HbSβ^+^  n = 25 | HbSS, HbSβ^0^  n = 38 |  |
| Age (yrs) | 31.25 (9.83) | 35.2 (11.21) | 37.08 (11.6) | ns |
| Sex (M/F) | 10/17 | 13/12 | 14/24 | ns |
| Hemoglobin (g/dL) | 13.37 (1.55) | 11.57 (1.77) | 9.33 (1.54) | a *vs.* c < 0.0001  b *vs*. c < 0.0001 |
| Hematocrit (%) | 40.55 (4.28) | 34.24 (5.26) | 27.53 (4.58) | a *vs*. b < 0.0158  a *vs*. c < 0.0001  b *vs*. c = 0.0002 |
| RBC count (M/µL) | 4.69 (0.49) | 4.24 (0.94) | 2.95 (0.67) | a *vs.* c < 0.0001  b *vs*. c < 0.0001 |
| WBC count (k/µL) | 6.82 (2.07) | 3.21 (0.64) | 10.64 (4.09) | a *vs.* c = 0.0006  b *vs*. c = 0.0133 |
| Platelet count (k/µL) | 244.6(48.0) | 285 (285.6) | 307 (93.7) | a *vs.* c = 0.0247 |
| MCV (fL) | 86.58(5.04) | 83.03 (13.6) | 94 .96 (11.43) | a *vs.* c = 0.0148  b *vs*. c < 0.0001 |
| MCH (pg) | 28.51 (1.96) | 28.12 (5.1) | 32.14 (4.10) | a *vs.* c = 0.0019  b *vs*. c < 0.0001 |
| MCHC (pg/mL) | 32.94 (0.69) | 33.79 (1.1) | 33.89 (0.93) | a *vs.* b = 0.0014  a *vs*. c = 0.0006 |
| RDW (%) | 13.9 (1.11) | 18.25 (3.03) | 19.65 (3.14) | a *vs.* b < 0.0001  a *vs*. c < 0.0001 |
| Retic count (M/µL) | - | 0.15 (0.12) | 0.27 (0.13) | < 0.0001 |
| Reticulocytes (%) | - | 3.02 (1.4) | 9.6 (5.1) | < 0.0001 |
| Ferritin (ng/mL) | - | 103.7 (111.2) | 1024 (1758) | < 0.0001 |
| %HbF | - | 5.8 (5.6) | 8.9 (8.26) | 0.1161 |
| %HbS | - | 44.43 (12.31) | 43.26 (25.44) | 0.96 |
| AST (U/L) | - | 25.33 (19.24) | 37.51 (28.53) | 0.0041 |
| Total bilirubin (mg/dL) | - | 1.167 (0.82) | 2.77 (2.19) | < 0.0001 |
| PNP (pg/mL) | 1310 (977.5) | 1681 (1205) | 3490 (3400) | a *vs.* c = 0.0001  b *vs*. c = 0.013 |
| LDH (U/L) |  | 249.6 (84.3) | 373 (146.7) | <0.0001 |

Data are mean (standard deviations). Analysis by Kruskal-Wallis test with Dunn’s posthoc test; Mann-Whitney test for unpaired parameters

***Association of plasma levels of PNP with markers of hemolysis in patients with mild and severe SCD***

The plasma levels of PNP positively correlated with plasma levels of LDH and negatively with the hemoglobin levels in patients with severe SCD, while other parameters were not significantly correlated. All parameters did not significantly correlate with the PNP levels in patients with mild disease (supplementary data Table s3).

**Table s3: Association between *plasma purine nucleoside phosphorylase (PNP) levels and markers of hemolysis in SCD subgroups***

| Parameters | Mild disease | | Severe disease | |
| --- | --- | --- | --- | --- |
|  | HbSC, HbS/β^+^  n = 25 | | HbSS, HbS/β^0^  n = 38 | |
| PNP levels *vs.* | **r (95% CI)** | **p-value** | **r (95% CI)** | **p-value** |
| Plasma LDH U/L | 0.34 (-0.08 - 0.66) | 0.0971 | 0.39 (0.06 - 0.64) | **0.019** |
| Hemoglobin (g/dL) | -0.28 (-0.60 - 0.13) | 0.1596 | -0.37 (0.62 - -0.05) | **0.022** |
| Platelets (K/Ul) | 0.24 (-0.17- 0.57) | 0.24 | 0.39 (0.067 - 0.64) | **0.017** |
| Reticulocytes (%) | 0.36 (-004 - 0.67) | 0.07 | -0.067 (-0.39 -0.27) | 0.69 |
| Ferritin (ng/mL) | -0.04 (-0.46 - 0.40) | 0.86 | 0.02 (-0.33 - 0.37) | 0.91 |
| Total bilirubin (mg/dL) | -0.19 (-0.6 - 0.26) | 0.39 | 0.05 (-0.29 - 0.38) | 0.76 |
| AST (U/L) | -0.15 (-0.5 - 0.03) | 0.4969 | 0.24 (-0.10 - 0.53) | 0.16 |

***8-AG inhibits PNP activity and rebalances dysregulated purine metabolism in SS mice***

Purine urinary metabolome showed that the PNP inhibition led to significant changes to the purine urinary metabolome in SS mice treated with 8-AG suggesting the protective effect of 8-AG in SS mice (Figure s1). In SS mice, 20-week treatment with 8-AG did not inhibit ADA, as evidenced by no changes in adenosine and inosine levels and adenosine/inosine ratio (A, B & E). 8-AG treatment significantly reduced the urinary levels of the direct products of PNP activity, hypoxanthine (**C**) and guanine (**I**), and increased the inosine/hypoxanthine (**F**) and guanosine/guanine (**J**) ratios suggesting an efficient PNP inhibition by 8-AG in the SS mice. Notably, in SS mice, 8-AG treatment also led to increased urinary levels of guanosine (**H**), a purine nucleoside with putative multifaceted beneficial effects in sickle cell disease (*see visual abstract)*.


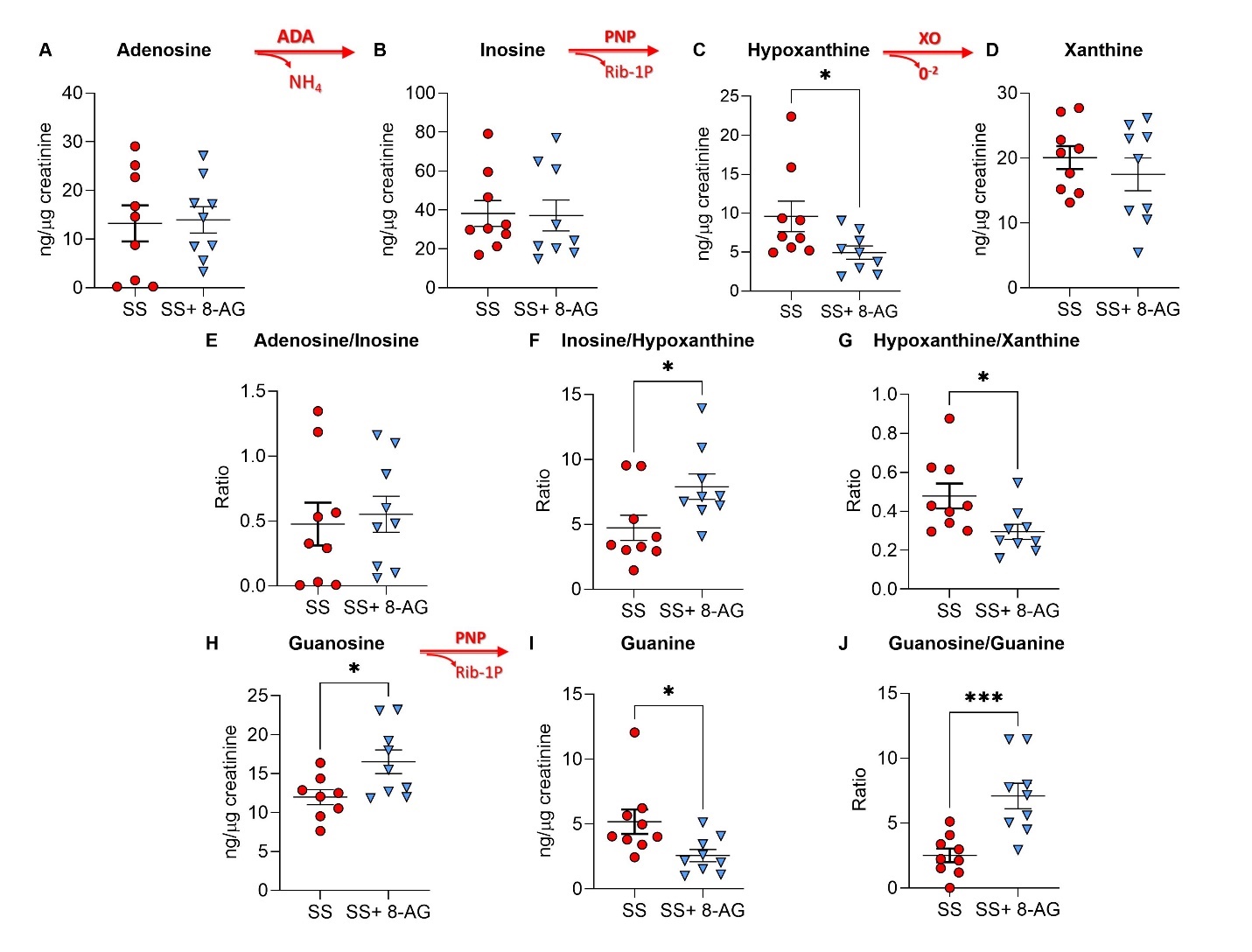


**Figure s1. Purine urinary metabolome in SS mice.** 20-week treatment with 8-AG did not affect urinary adenosine (**A)** and inosine (**B**) levels and the adenosine/inosine ratio (**E**) in SS mice, confirming that 8-AG does not inhibit ADA. 8-AG treatment reduced the urinary levels of the direct products of PNP activity, hypoxanthine (**C**), and guanine (**I**) with increased inosine/hypoxanthine ratio (**F**) in the SS mice suggesting an efficient PNP inhibition by 8-AG in the SS mice. Urinary hypoxanthine-xanthine ratio (**G**) was reduced in the 8-AG-treated SS mice suggesting an indirect reduction in XO activity by 8-AG due to reduced hypoxanthine and subsequently reduced downstream production of xanthine (**D**). Furthermore, 8-AG treatment led to increased urinary levels of guanosine, reduced guanine levels and increased guanosine/guanine ratio (**H, I, and J** respectively), all three are indicative of an efficient inhibition of PNP in SS mice. Arrows indicate the direction of the metabolic reaction. Purine nucleoside phosphorylase (PNP), adenosine deaminase (ADA), ribose-1-phosphate (Rib-1P); Townes SS mice (SS) and SS mice with 8-weeks of treatment with 8-aminoguanosine (SS+8AG). * p < 0.05, *** p < 0.001, unpaired t-test n = 11 per group.

***8-AG did not affect the RBC indices***

After 20 weeks, the untreated AA mice had lower MCV, MCH, and RDW, but there was no difference in MCHC (Figure s1). There was an initial decline in MCV, MCH, and MCHC after the initial 10 weeks of treatment, but this decline was halted thereafter.

**
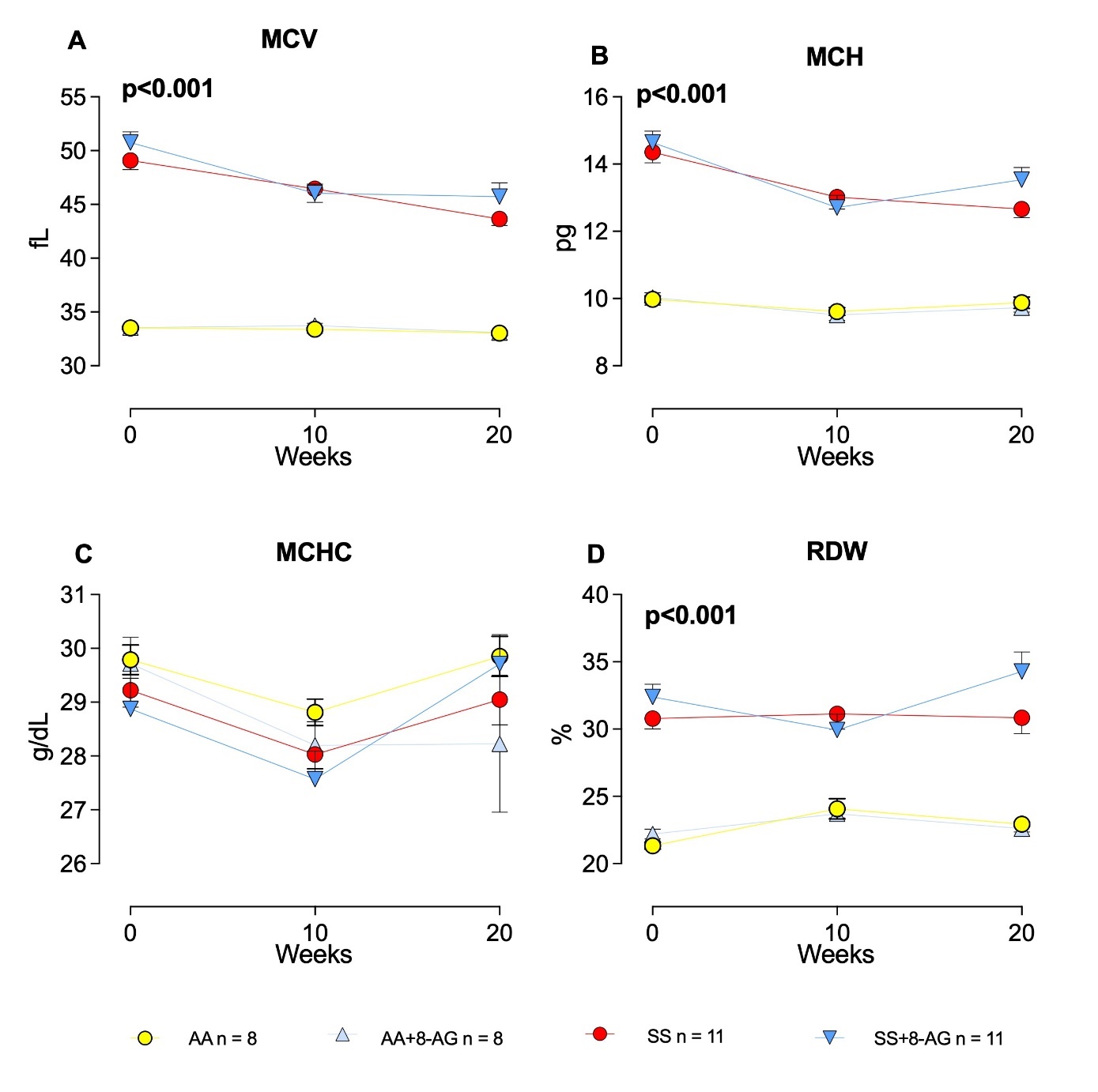
**

**Figure s2: RBC indices in Townes AA and SS mice treated with 8-AG or not treated**. Following 20 weeks, the untreated AA mice had lower mean cell volume (MCV) (**A**), mean cell hemoglobin (MCH) (**B**)**,** and lower red cell distribution width (RDW) (**D**)**.** There was no difference in mean cell hemoglobin concentration (MCHC) (**C**)**.** 8-AG halted the decline in the red cell indices after the initial 10 weeks of treatment. Townes SS mice without 8-AG treatment (SS) and Townes AA mice (AA) without 8-AG treatment, Townes SS mice with 8-Aminoguanosine (SS+8AG), Townes AA mice with 8-Aminoguanosine (AA+8AG). 2 factor ANOVA (2F-ANOVA).

***8-AG did not impact lung histology***

Sickle cell mice had significantly increased lung mass compared to AA mice, and treatment with 8-AG did not impact this change, Figure s3**.** There were no signs of pulmonary infarction and hemorrhages in both AA and SS mice, but the findings in the untreated SS mice showed increased mild emphysema and perivascular edema with increased presence of inflammatory cells and mild vascular remodeling. 8-AG did not affect lung histopathology.


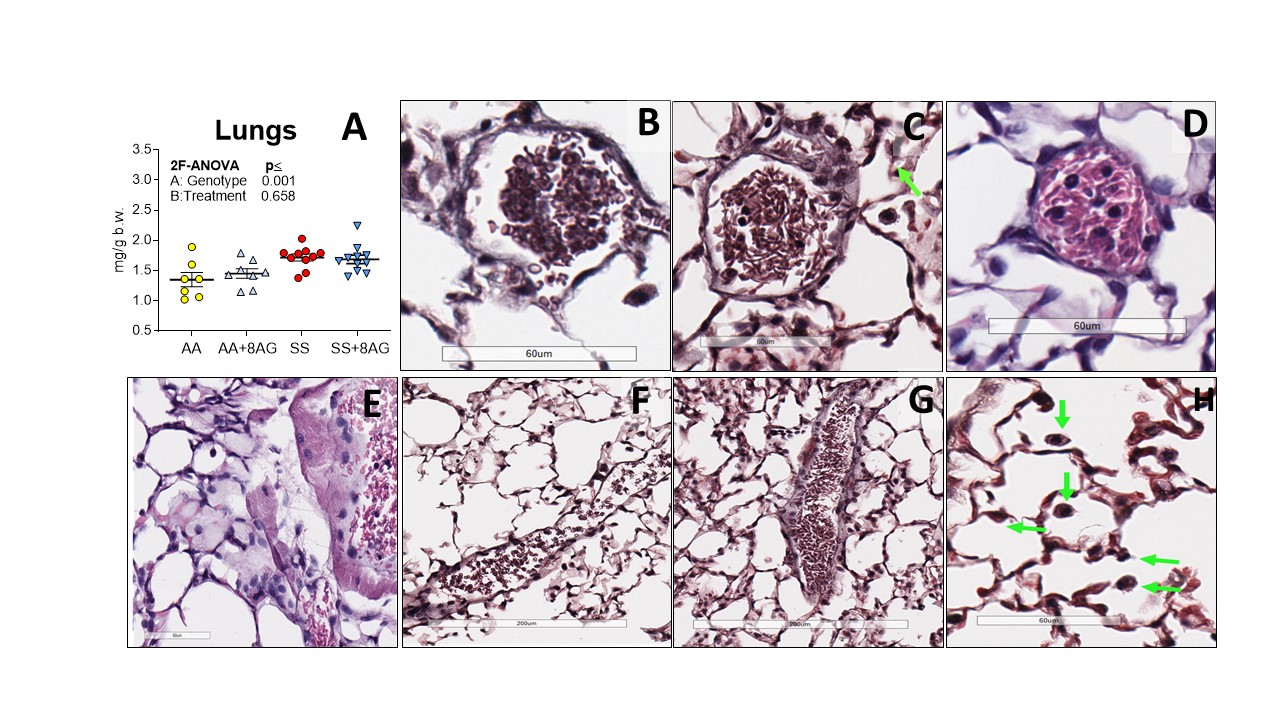


**Figure s3: Lung histopathology:** SS mice had increased lung weight compared to AA controls (genotype effect p < 0.001); (**A**). There were no signs of pulmonary infarct and hemorrhages in SS mice. Perivascular (**C**) and alveolar (**I**) presence of inflammatory cells, mild emphysema (**E & F**) and perivascular edema (**D**), and dilated and congested vessels with mild vascular remodeling (**D & G**) were seen in SS mice compared to AA mice (**B**). 8-AG did not affect lung histopathology. Green arrows = regions of perivascular (**C**) or alveolar space (**H**) macrophage or neutrophil infiltration; Pentachrome staining: Scale bars 60 µm.

***Townes SS mice exhibited LV hypertrophy and 20-week 8-AG treatment did not impact the LV function***

Using non-invasive echocardiography, we examined the effect of 8-AG on the left ventricle (LV) in both AA and SS Townes mice. Following 20 weeks of 8-AG or placebo, while the genotype did not significantly affect the body weights (p = 0.266), the 8-AG treatment significantly reduced the body weights (p = 0.004), Figure s4A. The left ventricle echocardiographic parameters (normalized by body weight) were indicative of LV hypertrophy and dilated cardiomyopathy in accordance with previous reports in SS Townes mice and SCD patients^1,5,6^. In this regard, the SS mice had increased LV mass (p = 0.001), LV internal diameter during systole (LVIDs, p = 0.002) and diastole (LVIDd, p = 0.002), LV posterior wall thickness during systole (LVPWs, p = 0.011) and diastole (LWPWd, p = 0.019), LV end-systolic (LV ESV p = 0.001) and end-diastolic volume (LV EDV, p = 0.001), LV stroke volume (p = 0.001), and cardiac output (p = 0.009) (Figures s4 B, C, D, G, H, I, J, K, and L, respectively). However, there was no difference in ejection fraction confirming previous reports that SCD mice exhibit features of HFpEF.^1,5^


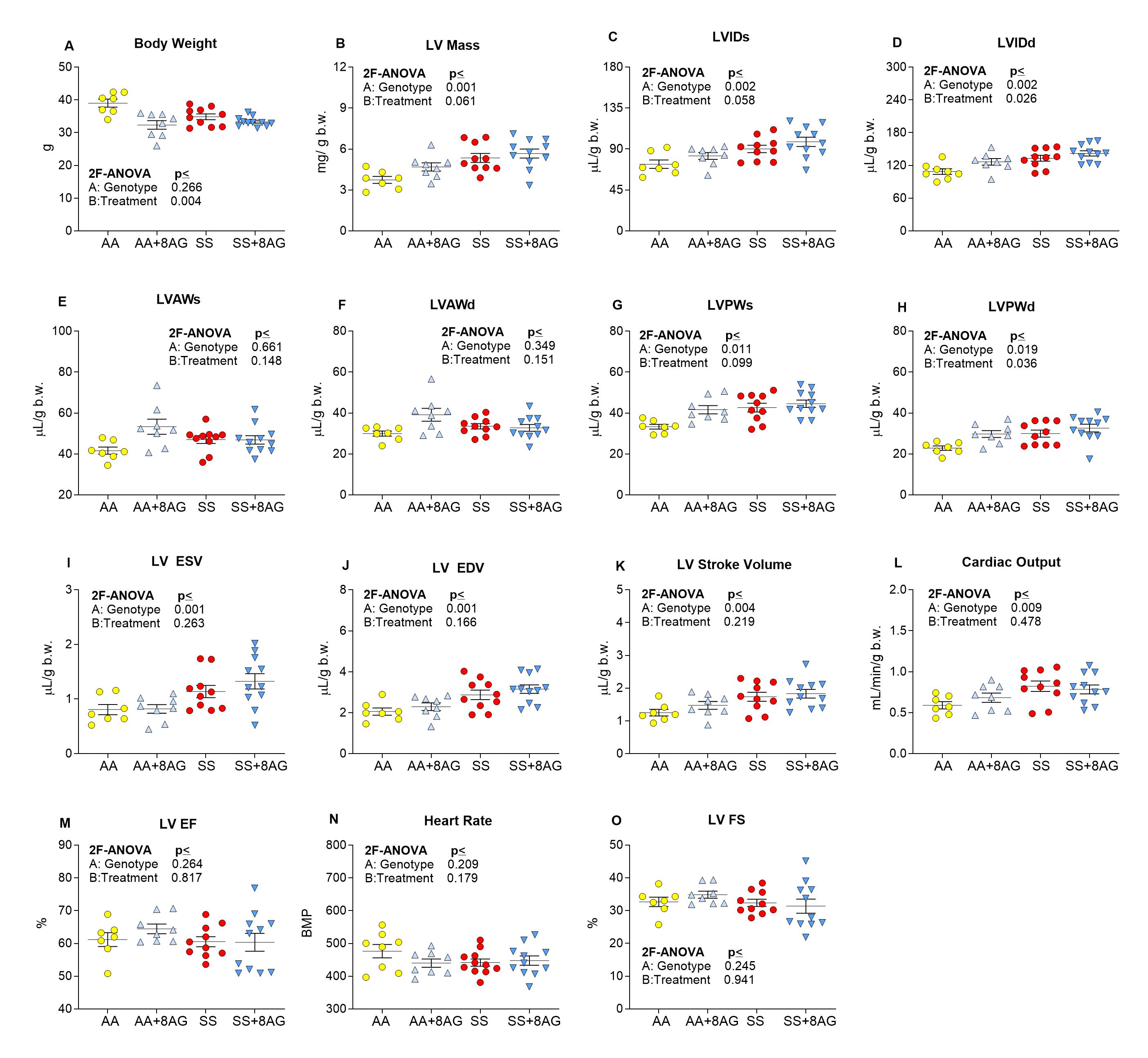


**Figure s4: Non-invasive echocardiography of left ventricle (LV) in Townes mice with placebo (AA and SS) or 8-aminoguanosine treatment (AA+8AG and SS+8AG) for 20 weeks.** The body weight was not significantly different between genotypes (**A**)**.** The SS mice had increased left ventricular (LV) mass (**B**), LV internal diameters during systole, LVIDs (**C**), LVID during diastole, LVIDd (**D**). Both genotypes and treatment did not affect the LV anterior wall during systole, LVAWs (**E**), and LV anterior wall during diastole, LVAWd (**F**)**.** The SS mice had higher LV posterior wall during systole LVPWs (**G**), LV posterior wall during diastole, LVPWd (**H**), LV end-systolic volume, LVESV (**I**), LV end-systolic volume, LVEDV (**J**), LV stroke volume (**K**), and cardiac output (**L**). The. genotype and 8-AG treatment did not impact the LV ejection fraction, LV EF (**M**), heart rate (**N**), and LV fractional shortening, LV FS (**O**). Townes SS mice (SS), Townes AA mice (AA), Townes SS mice with 8-Aminoguanosine (SS+8AG), Townes AA mice with 8-Aminoguanosine (AA+8AG), 2 factor ANOVA (2F-ANOVA); n = 7 – 10**.**

***8-AG reduced splenomegaly in Townes SS mice***

There was massive splenomegaly in the SS compared to the AA mice as expected, Figure s5. The SS mice had markedly distorted splenic architecture, and all animals had significant iron deposition in the spleen. The mice treated with 8-AG had reduced splenomegaly and tended to have less distorted splenic architecture.


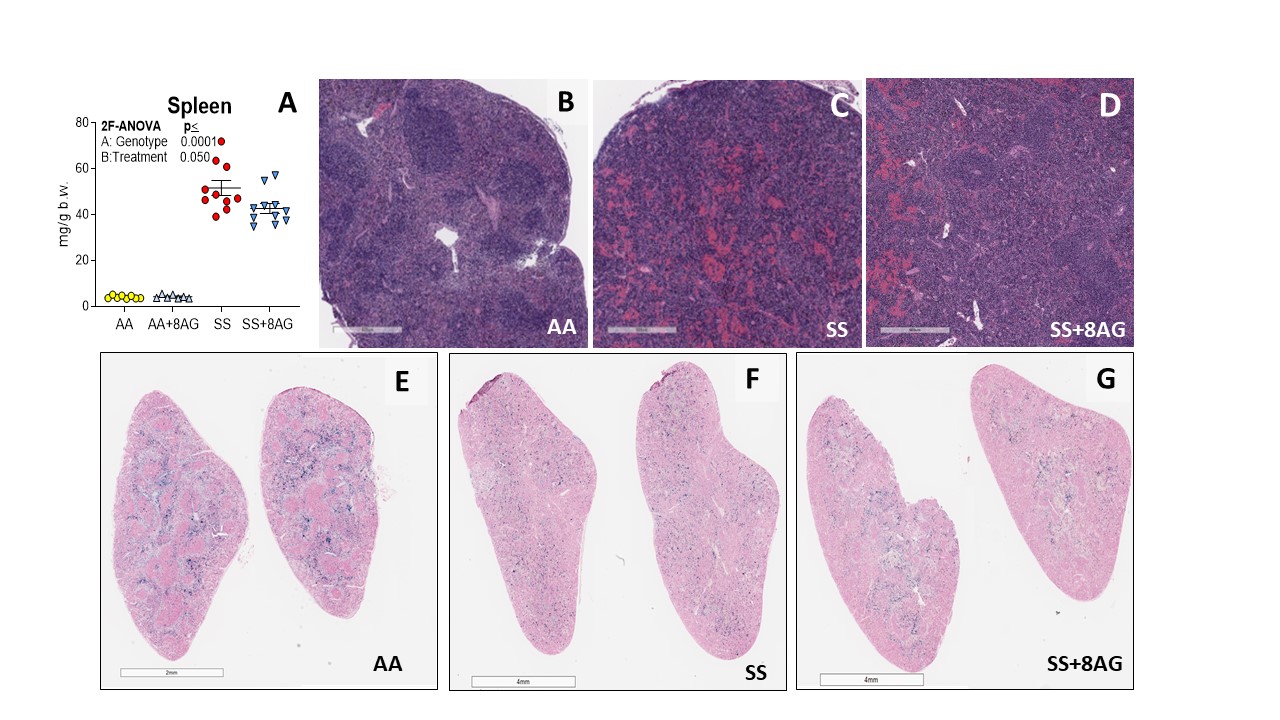


**Figure s5: Splenic histopathology:** SS mice exhibited massive splenomegaly (**A**) and, compared to AA controls (**B**) which have organized areas of white pulp and red pulp, the SS mice had markedly distorted splenic architecture (**C**). All mice groups had significant iron disposition (**E–G**). 8-AG reduced splenomegaly and tended to have less distorted splenic architecture (**D & G**). B - D: Hematoxylin and Eosin; E - G Prussian blue staining. Bar scale 2mm (B&E) and 4mm (C, D, F & G).

**References:**

1. Alvarez-Argote J, Dlugi TA, Sundararajan T, et al. Pathophysiological characterization of the Townes mouse model for sickle cell disease. *Transl Res*. 2023;254:77-91.

2. Rutledge C, Cater G, McMahon B, et al. Commercial 4-dimensional echocardiography for murine heart volumetric evaluation after myocardial infarction. *Cardiovascular Ultrasound*. 2020;18(1):9.

3. Potoka KP, Wood KC, Baust JJ, et al. Nitric Oxide-Independent Soluble Guanylate Cyclase Activation Improves Vascular Function and Cardiac Remodeling in Sickle Cell Disease. *American journal of respiratory cell and molecular biology*. 2018;58(5):636-647.

4. Hu J, Sharifi-Sanjani M, Tofovic SP. Nitrite Prevents Right Ventricular Failure and Remodeling Induced by Pulmonary Artery Banding. *J Cardiovasc Pharmacol*. 2017;69(2):93-100.

5. Bakeer N, James J, Roy S, et al. Sickle cell anemia mice develop a unique cardiomyopathy with restrictive physiology. *Proc Natl Acad Sci U S A*. 2016;113(35):E5182-5191.

6. Gladwin MT, Sachdev V. Cardiovascular abnormalities in sickle cell disease. *J Am Coll Cardiol*. 2012;59(13):1123-1133.
